# Husk Separation (Kubessa Method) Impacts the Aging Chemistry of Beer

**DOI:** 10.1021/acs.jafc.4c05099

**Published:** 2024-09-02

**Authors:** Stefan A. Pieczonka, Lukas Brass, Florian Lehnhardt, Jens Eiken, Alexa Wachtler, Leopold Weidner, John Brauer, Michael Rychlik, Martina Gastl, Philippe Schmitt-Kopplin, Martin Zarnkow

**Affiliations:** † Analytical Food Chemistry, TUM School of Life Sciences, Technical University of Munich, 85354 Freising, Germany; ‡ Analytical BioGeoChemistry, Helmholtz Association, Helmholtz Munich, 85764 Neuherberg, Germany; § Research Center Weihenstephan for Brewing and Food Quality, Technical University of Munich, 85354 Freising, Germany; ∥ International Flavors & Fragrances Inc. (IFF), 2800 Kongens Lyngby, Denmark; ⊥ European Brewery Convention (EBC), The Brewers of Europe, 1050 Brussels, Belgium

**Keywords:** beer, husk separation, aging, Maillard reaction, FT-ICR-MS, machine learning

## Abstract

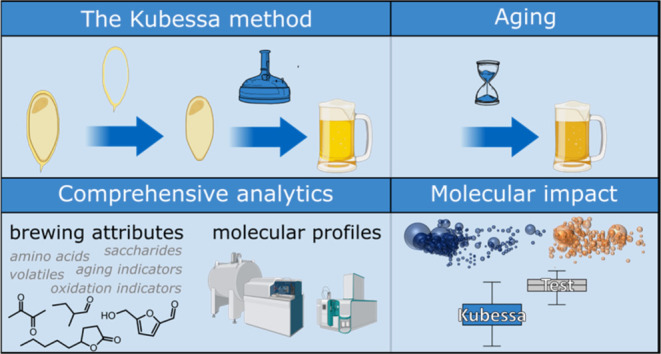

The removal of husks before the mashing process, also known as the Kubessa method, is an established brewing practice often positively associated with smoothness and better flavor-stability of beer. Empirical evidence on the effect of the Kubessa method on beer, however, has been lacking. Similarly, our study’s comprehensive analysis of established brewing attributes revealed that traditional methods do not fully capture the impact of husk separation in beer brewing. Conclusive evidence of the Kubessa method’s impact on beer aging chemistry was obtained through ultrahigh resolution mass spectrometry (FT-ICR-MS), revealing intricate molecular details inaccessible to conventional analytical techniques. The compositional information on thousands of molecules in Kubessa beer was resolved and compared to whole malt mashing. Machine learning algorithms applied to aging experiments identified over 500 aging-related compounds inhibited by husk separation. Complementary Time of flight mass spectrometry (ToF-MS) coupled with chromatography further confirmed that the mashing of husks introduces sulfur-containing lipid compounds. These significant differences in the beer composition provide valuable insights for further investigation into the staling protective effect of husk-separation (Kubessa process) during beer production, as empirically demonstrated in this work.

## 1 Introduction

The brewing industry constantly investigates specialized processes to improve beer quality and sensory characteristics. The Kubessa method, developed by Richard Kubessa in the early 20th century,^1^ is distinguished by an innovative mashing approach that produces beers with improved smoothness and extended shelf life. This mashing technique involves the preliminary separation of the barley husk from the starchy endosperm of the malt grain, with the husk being reintroduced at a later stage of the mashing process. It is designed to minimize the leaching of husk compounds as their absence is intended to enhance the beer’s flavor. Although the underlying mechanisms are still poorly understood, this process is believed to be crucial in improving the beer’s overall quality, smoothness, and longevity.

Beer aging (also interchangeably referred to as beer staling) is a complex phenomenon that leads to changes in the sensory properties of beer over time. This process is influenced by a myriad of chemical reactions that occur during storage, changing the chemical composition of the beer and, consequently, its quality.^2^ The primary focus of beer aging research has been to understand the formation of specific staling compounds, such as (*E*)-2-nonenal, which is known to impart a cardboard flavor through lipid oxidation. For practical reasons, the complex network of Maillard reactions is simplified by parameters such as EBC^3^ or MEBAK^4^ methods, thiobarbituric acid-reactive substances, or hydroxymethylfurfural (HMF) as an indicator compound. In brewing science, the introduction of advanced analytical methods has enabled the elucidation of the extensive variety of Maillard compounds,^5^ facilitating a detailed investigation of the influence of brewing parameters on their formation and prevalence.

Compounds derived from the husk are thought to influence a number of metabolites associated with the formation of stale flavors in beer. The presence of excess organic radicals and iron in the husk,^6^ coupled with the leaching of polyphenolic compounds,^7^ is thought to be detrimental to shelf life. However, the specific molecular changes induced by husk-derived compounds during beer aging remain to be elucidated.

Our study leverages a comparative analysis of beers brewed with and without the Kubessa method, using advanced analytical techniques to investigate the molecular aspects of beer stalling. By identifying metabolites that differ significantly between the two brewing lines, we aim to shed light on the speculated benefits of the Kubessa method and provide empirical insights into its impact on the aging chemistry of beer.

## 2 Materials and Methods

### 2.1 Brewing and Aging Experiments

The brewing trials were conducted at the Distelhäuser Brauerei Ernst Bauer GmbH & Co. KG in Tauberbischofsheim, Germany, in 2021. Pilsner beer was brewed in 185 hl batches for the study. The experimental design included two brewing variants, which differed by their method of grain incorporation during the mashing process. Both methods start with milling the malt in a six-roll mill, where the husks are removed with sieves after they have been ground out. One variant used the Kubessa or husk separation method (Kubessa, K), where the removed husks were not part of the mash. They were only added during the final saccharification rest to ensure a good runoff during lautering. In the other variant, the whole grist load was used for mashing (Test, T). After lautering, the wort was boiled and hopped. The hot break was removed in a whirlpool. The cooled wort was separated from the cold break and aerated in a flotation tank. This combined three wort batches into one pitching wort. The main fermentation was carried out in a cylindroconical fermentation tank. When attenuation was 0.7% above the attenuation limit, the beer was transferred to horizontal storage tanks and stored at 3 °C for at least 4 weeks.

The beers were filtered using a sheet filter equipped with depth filter sheets. The filtered beers were then filled into 0.5 L bottles using a fully automated filler. To displace air from the bottleneck and facilitate proper sealing, the beers were gently tapped against the bottles to cause the release of CO_2_ and create foam before being promptly crown-corked. In between analyses, the beers were stored in a refrigerated chamber at 2 °C. The experimental design resulted in three test beers T using whole malt and one control beer K using the Kubessa method. All beers are based on three different mash replicates.

These beers were naturally aged at 2 °C in the dark for durations of two (*t*1), four (*t*2), and seven (*t*3) months. In addition, the beers were forced aged (forced-aged fo; 1 day overhead shaking, 70 rpm followed by 4 days storage at 40 °C). The brewing process, including the mash, sweet wort, boiled wort, young beer, and mature beer, was systematically sampled to assess the reproducibility and consistency of the brewing experiments and to trace possible differences throughout the process. An overview of the sample nomenclature is given in Supporting Table S1.

### 2.2 Established Malt, Wort, and Beer Attributes

The malt attributes extract (MEBAK^4^ R-205.01.080 [2016–03]), soluble nitrogen (MEBAK R-205.11.030 [2016–03]), thiobarbituric acid number (MEBAK R-205.21.111 [2016–03]) and pH value (MEBAK R-205.06.040 [2016–03]) were analyzed (*n* = 1). The other attributes are amino acids (MEBAK B-400.13.133 [2020–10] adjusted for malt), FAN (calculated from amino acid content), sulfite (modified MEBAK B-590.37.137 [2020–10]), and sugars (modified MEBAK B-590.12.134 [2020–10]) completed the analysis portfolio (*n* = 3). All these analyses were performed in our accredited laboratory at the Research Center Weihenstephan for Brewing and Food Quality following the internationally harmonized protocols of the Central European Brewing Technical Analysis Commission (MEBAK).

Similarly, the mash and beer attributes were analyzed using the respective MEBAK^4^ (Mitteleuropäische Brautechnische Analysenkommission) methods. The portfolio was extended for alcohol content (MEBAK WBBM 2.9.6.3), apparent extract (MEBAK WBBM 2.9.6.3), apparent attenuation (MEBAK WBBM 2.9.6.3), steam-volatile aroma compounds (MEBAK WBBM 2.23.6), volatile fermentation byproducts (MEBAK WBBM 2.21.1) and aging indicators (MEBAK B-420.24.151).

Sensory analysis was performed in an expert consensus panel with 4 trained and certified panelists. The 5-point DLG scheme was used. The quality of five categories (odor, taste, palate fullness, effervescence, and quality of bitterness) was rated on the following scale: 0 = inadequate (not detected); 1 = not satisfactory (substantial deviations); 2 = less satisfactory (apparent deviations); 3 = satisfactory (perceptible deviations); 4 = good (slight deviations); 5 = very good (quality expectations reached in full). The results are expressed in an overall DLG-score (DLG-score = (odor × 2 + taste × 2 + palate fullness + carbonation + quality of bitterness × 2)/8). The data can be found in the Supporting Information (Supporting Table S2) if not shown in the following lines.

### 2.3 FT-ICR-MS Metabolome Analysis

The brewing process and beer aging samples were subjected to solid phase extraction (SPE) prior to injection into the ultrahigh resolution mass spectrometry (FT-ICR-MS) system. The SPE attributes, reagents, measurement, and data processing attributes were selected as previously reported.^8,9^ The eluate was centrifuged, and the supernatant was used for metabolite analysis on a Bruker solariX Ion Cyclotron Resonance Fourier Transform Mass Spectrometer (Bruker Daltonics GmbH, Bremen, Germany) equipped with a 12 T superconducting magnet (Magnex Scientific Inc., Yarton, GB) and an APOLO II ESI source (Bruker Daltonics GmbH, Bremen, Germany) operated in negative ionization mode, accumulating 400 scans in a 10 min measurement. The negative ionization mode was chosen to avoid ion suppression of metabolites caused by dominant adducts of saccharides with sodium and potassium ions in ESI-positive direct infusion mass spectrometry. The mass resolving power was stable at 400,000 at *m*/*z* 400, and 81% of all detected monoisotopic signals could be assigned to a molecular formula within an average mass error range of ±0.2 ppm and a signal-to-noise ratio of 6. Signals occurring in at least 2 out of 3 replicates were considered, and intensity values were averaged. The merged feature matrix yielded a total of 4777 signals with unique, unambiguous molecular formulas covering the elemental space of CHNOSPCl and the mass range of *m*/*z* 120 to 1000.

### 2.4 LC-ToF-MS Metabolome Analysis

After SPE treatment, the brewing process and beer aging samples were analyzed using an AB Sciex X500R QTOF system (Darmstadt, Germany) coupled to an ExionLC UPLC system, employing data-dependent acquisition in negative ionization mode. The chromatographic and instrument parameters are detailed in the Supporting Information (Supporting Table S3). The manufacturer-specific.wiff2 files were converted to mzML files with the msconvert software (ProteoWizard)^10^ prior to further data processing in mzMine3.^11^ The data processing parameters for mass detection, smoothing, feature detection, and peak alignment are detailed in the Supporting Information (Supporting Table S4). Only signals detected in at least 2 out of 3 replicates were considered. The feature list, consisting of 1505 (aging experiments) and 3041 (brewing process) chromatographic features, was exported as an MFG file and subsequently analyzed within the Sirius 5.8.2 software environment.^12^ The analysis used tandem mass spectrometry data to determine molecular formulas,^13^ as well as to classify compound classes using CANOPUS^14,15^ and to generate structural suggestions via CSI:FingerID.^16^ The databases consulted, and the parameters applied in this process are detailed in Supporting Table S5.

### 2.5 Statistical Data Analysis and Data Visualization

#### 2.5.1 Established Malt, Wort and Beer Analyses

Data was analyzed using JMP Pro 17 (SAS Institute Inc., Cary, NC). Means and standard deviations were calculated from technical and biological replicates. One-way analysis of variance (ANOVA, α = 0.05) was used to determine statistical differences where indicated.

#### 2.5.2 Identification of Aging-Related Molecular Compositions (FT-ICR-MS)

Clustering of compounds based on their observed intensity profiles during the aging experiment was performed using a self-organizing map (SOM) implementation^17^ on the FT-ICR-MS data of the aging experiments at the Research Center Weihenstephan for Brewing and Food Quality (B_). Features that showed a consistent increase in intensity with aging in both beer lines (T and K) were defined as potential aging compounds. The molecular compositions of these compounds were visualized in van Krevelen diagrams, and by plotting their H/C against O/C elemental ratios, the proposed compound classes were determined.^18^ Using box plots for visualization, we used time-resolved and aggregated relative intensity values to compare the concentrations of aging-related compounds in the aged beers from the Kubessa brewing line with those from traditional mashing.

#### 2.5.3 OPLS-DA of the Differently Mashed Beers (FT-ICR-MS and LC-ToF-MS)

A supervised OPLS-DA analysis was performed on both the FT-ICR and UPLC-ToF data sets of the final beer samples (B_T_t0_R1 to B_T_t3_R3 and B_K_t0_R1 to B_K_t3_R3, respectively). In addition, a second set of the same beers, sampled at the time of sale of the beers (D_T and D_K), was used to increase the statistical power of the model. Hotelling’s *T*^2^ test (95%) was used to eliminate the influence of strong outliers on the models. The goodness of the fit and the prediction were assessed using the *R*^2^ and *Q*^2^ values. To rule out overfitting, we provide the *p*-value of the Cross-Validation Analysis of Variance (CV-ANOVA). These elaborations were carried out using the ropls package (R 4.1.2) within the RStudio environment (version 2023.12.0). Drawing from Chong and Jun,^19^ we employed a stringent VIP threshold of 2 for the most significant features in our OPLS-DA models to mitigate the risk of overfitting and enhance feature selection reliability in our high-dimensional data set, where the number of metabolites greatly exceeded the sample size from a single experimental trial. The potential markers for mashing with the Kubessa method were visualized in van Krevelen diagrams (FT-ICR-MS), and their compound class and structure were identified at level 3 of the confidence level in metabolomics^20^ (LC-TOF-MS), respectively.

## 3 Results and Discussion

### 3.1 Established Brewing Analyses

We investigated the potential impact of dehusking on classical brewing attributes through a comprehensive analysis of the amino acid distribution, free amino nitrogen (FAN), soluble nitrogen, saccharide distribution, extract, original gravity, color, attenuation, pH value, sulfite and dimethyl sulfide content, alcohol content, as well as measures and molecular indicators for thermal and oxidative stress, alongside sensory characteristics. This thorough approach allows us to capture potential effects at all levels of the multifaceted and complex brewing process, from the malt to the resulting beer.

#### 3.1.1 Malt Analysis

Laboratory mashing trials were carried out using 50 g of material per trial. It is important to note that the use of husked (Test T) or dehusked (Kubessa K) malt significantly affects the laboratory values, which are primarily influenced by the endosperm. Consequently, while it is possible to infer the origin of the compounds measured, the direct applicability to operational mashing processes is limited due to the consistent malt mass and the delayed addition of husks, resulting in a reduction in compound leaching.

The higher concentrations observed in huskless malt mashes are particularly striking in the sugar analyses of laboratory mashes. In particular, the maltose content in mash K is elevated (T: 34.8 g/L; K: 42.5 g/L), while other sugars also show higher levels but remain within the measurement uncertainty.

Conversely, amino acid concentrations were consistently higher in mashes containing husks, except those below the detection limit (aspartic acid, serine). Overall, mashes containing husks showed a 33.6% increase in amino acids (T: 103.98 mg/100 mL; K: 77.85 mg/100 mL). The most significant differences were observed for asparagine (T: 2.76 mg/100 mL; K: 1.30 mg/100 mL) and the nondetectable amino acids (glutamine, arginine, and histidine) in malt K.

The soluble nitrogen content was slightly higher (4.3%) in mashes containing husks, indicating a specific contribution of the husks to the amino acid concentration rather than the total nitrogen content.

The thiobarbituric acid number was also increased in conventional test mashes (Test T: 10.96; Kubessa K: 8.48), suggesting increased thermal stress on the husks during kilning. This may contribute to the perceived smoothness and shelf life of beers produced using the Kubessa method. Notably, despite these variations, no significant difference in the measured extract was observed between the experimental conditions.

Furthermore, the sulfite concentration varied between the different parts of the grain. The total malt grain showed a 15.5 mg/kg concentration, whereas the husk contained only 7.0 mg/kg. The highest concentration was found in the dehusked grain (16.2 mg/kg) - where sulfite is presumably present in the aleurone layer.

#### 3.1.2 Beer

Working with industrial-scale brews led to unforeseen restrictions during the pandemic backdrop. As a result, K needed to be assessed with a single combined biological triplicate (combination of three mashes in one fermentation tank and, therefore, one resulting beer). At the same time, T was evaluated with three distinct biological replicates for quantitative analyses. Standard beer analysis showed no significant differences between the different mashing methods (see Table 1). Only the content of soluble nitrogen was slightly elevated in T.

**Table 1:** Standard Beer Analyses

type of mashing	T	K
original gravity [mas %]	11.89 ± 0.08	11.86
alcohol content [vol %]	5.00 ± 0.02	5.01
apparent extract [mas %]	2.48 ± 0.05	2.43
apparent attenuation [%]	79.9 ± 0.3	80.3
pH-value	4.54 ± 0.01	4.55
soluble nitrogen [mg/100 g]	85.8 ± 0.4	83
thiobarbituric acid number (TBZ)	34.4 ± 0.7	34.1

A one-way ANOVA revealed differences in amino acid concentrations between samples K and T, with the husked beers (T) exhibiting a trend toward higher total amino acid content (T: 126.5 mg/100 mL; K: 119.0 mg/100 mL). Statistically significant differences (α = 0.05; *p* < α) were obtained for valine (*p* = 0.005), methionine (*p* = 0.004), isoleucine (*p* < 0.0001), phenylalanine (*p* = 0.04), leucine (*p* = 0.003) and lysine (*p* = 0.02).

Conversely, as the literature suggests,^21^ selected aging indicators were not elevated in T and did not show consistent differences between variations as indicated by the mean values over all aging points (3-methylbutanal (K: 14.2 ± 1.6 μg/L; T: 14.1 ± 0.9 μg/L); furfural (K: 62.5 ± 42.3 μg/L; T: 72.6 ± 24.4 μg/L); γ-nonalactone (K: 26.4 ± 3.9 μg/L; T: 25.9 ± 2.2 μg/L); phenylacetaldehyde (K: 6.9 ± 1.6 μg/L; T: 5.3 ± 0.9 μg/L)). Furthermore, no significant differences in aroma compounds or sugars were found between the brews (Supporting Table S2).

#### 3.1.3 Sensory Evaluation

Both variations showed a course of aging, characteristic of the beer styles, discovered with sensory analysis. The overall DLG-score shows a continuous decrease. After four months, the first signs of aging were detected in the consensus-panel. In the seven months stored samples, bread-like aromas were observed. In the overall DLG-score, the forced aged samples resembled 4 to 7 months of natural aging. Yet, no statistically significant differences were found between the two variations. The sensory data can be found in Supporting Table S2.

The Kubessa method represents a brewing technique that has been empirically established for decades. Yet, its application has not yet been justified by its effect on beer attributes or molecular composition. Similarly, our analyses using conventional brewing technology have revealed only minor differences, notably in increased maltose and total amino acid levels. Moreover, classical indicators of aging have not shown significant distinctions. Additional analyses beyond classical brewing analytics are required to empirically prove the staling protective effect of dehusking. As already demonstrated for aging indicators,^9,22^ alternative methods are often necessary to gain deeper insights and enable more meaningful interpretations. Therefore, in the following, we expand our focus beyond conventional aging markers by employing comprehensive nontargeted FT-ICR-MS analysis capable of resolving thousands of molecular descriptors.

### 3.2 Impact on the Aging Chemistry (FT-ICR-MS)

**Figure 1:**
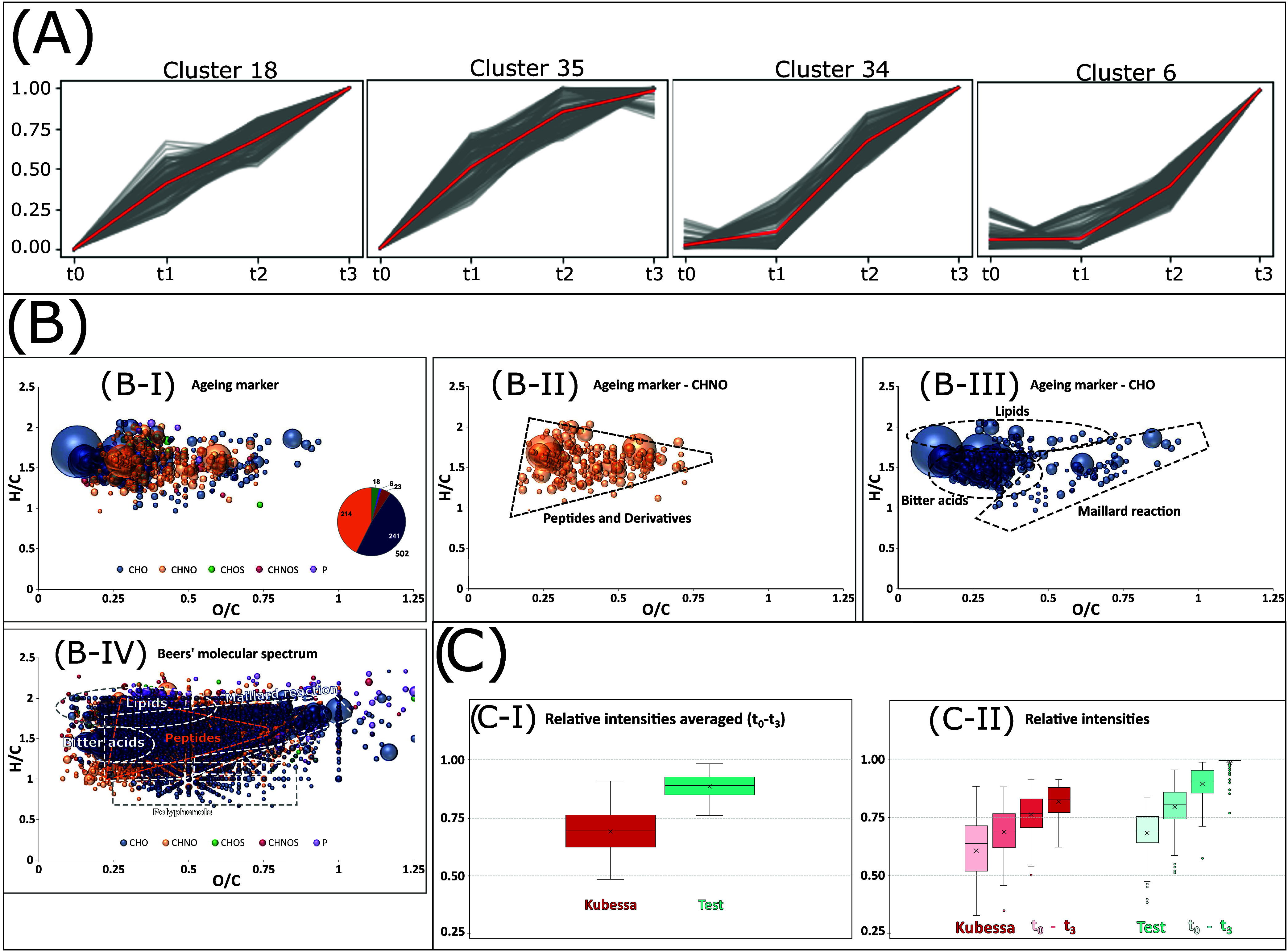
Machine learning extraction (A) visualization (B) and intensity value comparison (C) of aging-related compounds. Features that exhibit a similar intensity trend over the course of aging were categorized into clusters using a self-organizing Map (SOM) analysis. Four of these clusters, exhibiting consistent behavior across both brewing lines, are presented in (A). The complete SOM data processing can be found in Supporting Figure S1. In (B), the van Krevelen diagrams of the corresponding aging-related compounds are displayed in their entirety (B–I), molecules with CHNO in their formula (B–II), those with only CHO (B–III), and the entire beer for comparison (B–IV). In (C), the relative intensities of the features in both brewing lines are averaged across all time points (C–I) and presented as time-resolved (C–II) in box plots.

Following aging for two (*t*1), four (*t*2), and seven (*t*3) months, the beer samples (Supporting Table S1) were analyzed using ultrahigh-resolution mass spectrometry (FT-ICR-MS). The self-organizing map (SOM), an unsupervised machine learning technique, was utilized to identify aging-related molecular changes from the comprehensive molecular profile. In this way, we identified 502 molecular compositions that increased in intensity over the storage period in both beers (K and T) (Figure 1A). To further characterize these compounds, we exploited the crucial advantage of ultrahigh resolution, which allows for determining accurate masses and, consequently, molecular formulas. The van Krevelen diagrams (plotting H/C against O/C ratios) reveal that these specific compounds cluster in distinct regions (Figure 1B–I to III), contrasting with the broad molecular distribution observed for the whole beer matrix^23,24^ (Figure 1B–IV).

Through separating the compositional spaces of CHO (Figure 1B–II) and CHNO (Figure 1B–III), the compound classes of these molecules become evident: Our analytical and statistical approach has uncovered over 500 compounds that have mainly formed by oxidation (oxidized lipids and hop-derived compounds) and through Maillard chemistry^5^ (amino acid conjugates of sugars and their derivatives) during storage. These compounds are produced by well-known chemical reactions during beer aging^2^ and were notably present in previous FT-ICR-MS studies of a historical beer with extensive storage time.^9^ The intensity values of these aging-related molecules are less pronounced in the replicates of Kubessa beers compared to the beer samples where the husk was included in the mashing process (Figure 1C–I). The time-resolved analysis shows that husk separation’s effect goes beyond beer’s chemical aging during storage. The finished beers already exhibit reduced levels of these components at the start of the storage experiments (*t*0) (Figure 1C–II). It can, therefore, be concluded that the protective effect of the Kubessa method is already manifested during the brewing process itself. The progression over the storage period, from time *t*1 to *t*3, shows a similar trend in both brewing lines. However, the introduction of husk separation appears to have a mitigating effect.

We comprehensively investigated the Kubessa method’s impact on beer’s aging chemistry using ultrahigh resolution mass spectrometry and machine learning techniques. A decrease in 500 molecular compositions related to oxidation and Maillard chemistry highlighted the significant protective effect of husk separation in mitigating the molecular changes associated with beer aging. This finding is not limited to beer storage alone but manifests its impact during brewing.

### 3.3 Differences in the Beers’ Molecular Composition (FT-ICR-MS)

To investigate the cause of the different behavior of the beers during storage, the FT-ICR-MS compositional data of the finished beers were analyzed using Orthogonal Projection to Partial Least Squares Discriminant Analysis (OPLS-DA). The supervised statistical analysis included beers from the storage experiment (B_) and beers bottled from the Distelhäuser brewery (D_). An R2X value of 0.5 suggests that the whole-malt and Kubessa brewing lines are fundamentally very similar and were brewed with high reproducibility, with a significant proportion of the variance attributable to the distinction between Kubessa and whole-malt mashing. The goodness of fit (R2Y 0.97) and quality of prediction (*Q*^2^ 0.57) values were significant. The score plot, loadings plot, and variable of importance for prediction (VIP) values are shown in Supporting Figure S2.

**Figure 2:**
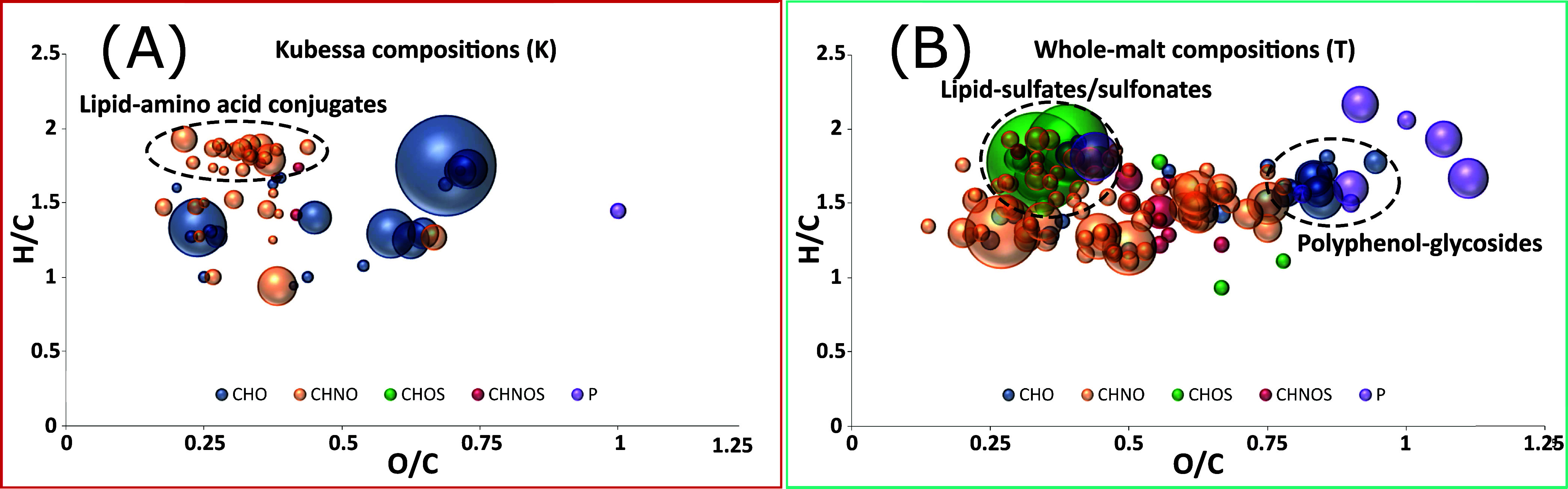
Van Krevelen representation of the compounds specific for the Kubessa method (A) and whole-malt mashing (B), respectively. Cluster of compound classes are highlighted.

The van Krevelen representation of compounds significant to the Kubessa method shows a clustering of potential amino acid conjugates of lipids (Figure 2A). The hypothesis that husk separation affects lipid metabolism, as suggested by van Waesberghe,^7^ is supported by a variety of lipid sulfates or sulfonates leached from the husk. Similarly, signals indicative of a molecular composition related to polyphenolic glycosides are specific to husk mashing (Figure 2B). Matching the *in silico* deglycosylated masses with database entries supports this hypothesis. Due to the poor ionization of polyphenols in ESI-negative mode, a definitive statement regarding the corresponding free aglycones cannot be made.

Despite the exceptional capabilities of ultrahigh resolution FT-ICR-MS, it provides structural information limited to atomic compositions. To further characterize compounds associated with the staling-protective effect of the Kubessa method, we employed complementary UPLC-ToF-MS with tandem mass spectrometry MS^2^ capabilities.

### 3.4 Confirmation of the Husk-Related Sulfur-Containing Compounds (UPLC-ToF-MS)

We used Time-of-Flight (ToF) mass spectrometry coupled to chromatography as a complementary analytical method to obtain structural information beyond the compositional space of isomer separation and tandem mass spectrometry. MS^2^ data were obtained for 894 (60%) of the total 1505 chromatographic features in the DDA acquisition. Even at the stage of the unsupervised PCA analysis stage, a discernible divergence between the two beer types was observed (Supporting Figure S3). Analogous to the FT-ICR-MS data, the supervised OPLS-DA of the UPLC-ToF-MS data yielded significant statistics with meaningful R2Y (0.99) and Q2 (0.88) values (Supporting Figure S3).

The most significant compounds coincided with the sulfur-containing compositions identified in the FT-MS measurements, as determined by the formula assignment of the fragmentation spectra^14^ (Table 2).

**Table 2:** Mass-to-Charge Ratios (m/z), Retention Times (RT), Molecular Formula and Compound Class Annotated in Sirius and FT-ICR-MS, and Notable Tandem Mass Spectrometric Ions/Neutral Losses of Compounds Most Significant (VIP > 2.5) for Mashing with Husk^a^

*m*/*z*	RT [min]	formula	compound class	FT-ICR-MS	notable ions|neutral losses
375.1832	5.99	C_18_H_32_SO_6_	sulfonate	C_18_H_32_SO_6_	[SO_2_]^−^|[SO_3_]^−^|H_2_O SO_3_
403.2152	8.32	C_20_H_36_SO_6_	sulfonate	C_20_H_36_SO_6_	[SO_2_]^−^|[SO_3_]^−^
389.1631	5.89	C_18_H_30_SO_7_	sulfonate	C_18_H_30_SO_7_	[SO_3_]^−^|H_2_O
448.2519	8.34	n.a.	n.a.	n.a.	n.a.
191.0742	6.21	C_8_H_16_SO_3_	sulfonate	C_8_H_16_SO_3_	[C_2_SO_3_]^−^
379.1759	4.54	n.a.	n.a.	C_20_H_28_O_7_	n.a.
264.0547	0.91	C_9_H_17_NO_7_S	sulfonate	C_9_H_17_NO_7_S	n.a.
389.1992	7.59	C_19_H_34_SO_6_	sulfonate	C_19_H_34_SO_6_	[SO_2_]^−^|[SO_3_]^−^
373.1681	5.87	C_18_H_30_SO_6_	sulfonate	C_18_H_30_SO_6_	[SO_2_]^−^|[SO_3_]^−^|H_2_O SO_3_

a The tandem mass spectra can be found in Supporting Figure S4.

**Figure 3:**
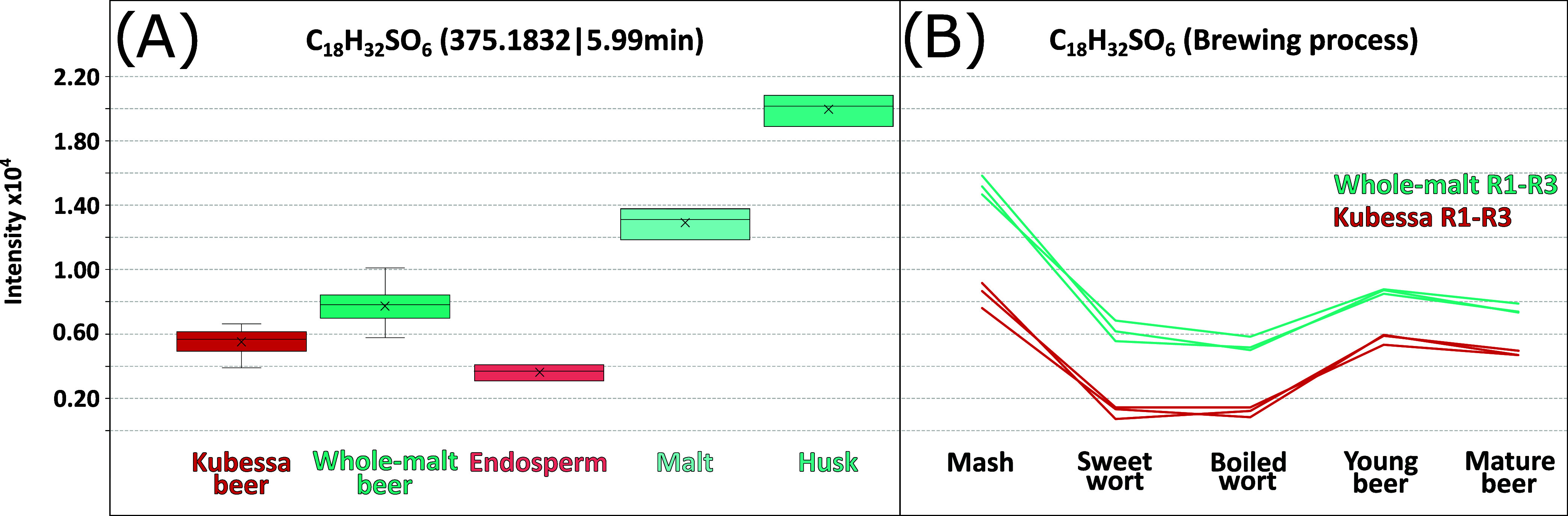
Intensity of the C_18_H_21_SO_6_ metabolite in the Kubessa beer, whole-malt beer, the malt grain compartments (A), and throughout the brewing process (B).

The LC-ToF-MS measurements confirm that the content of sulfur-containing lipids is increased in whole malt mashing and that their incorporation can be reduced by the Kubessa method (Figure 3A). A comparison of their content in malt, endosperm, and husk (20 °C isothermal mash) strongly points to the husk as the primary source of these compounds. The intensity trend is parallel in both beers during the brewing process, indicating that the difference in concentration is already established during the mashing process (Figure 3B).

Specific common neutral losses and fragmentations provide further insight into the structure and oxidation state of sulfur (Supporting Figure S4): in particular, a bisulfate anion (HSO_4_^–^, 96.9601) does not appear in any tandem mass spectra. In collisionally induced dissociation, this ion would be formed when a sulfate group is cleaved in a cyclic syn-elimination; a reaction that is only possible when a potential sulfate is bound aliphatically^25^ rather than phenolically. Given the high saturation level of the compounds, an aromatic moiety and therefore a sulfate group is unlikely. The presence of SO_2_^–^ and SO_3_^–^ fragments confirm the plausible presence of a sulfonate over a sulfate group. Our conclusions are confirmed by the sulfonate compound class annotation in Sirius-CANOPUS^15^ (Table 2). Therefore, the tandem mass spectrometry data indicate sulfonated lipids such as 18:2(OH)(SO_3_) or HODE-SO_3_ (C_18_H_32_SO_6_) and 20:3(OH)(SO_3_) or HEDE-SO_3_ (C_20_H_36_SO_6_). Detailed structural determination and identification of these compounds on identification level 1^20^ will require more targeted analysis and isolation or rather synthesis in further studies and specialized brewing experiments to substantiate the correlative relationship we found with potential causal mechanisms.

In conclusion, our comprehensive analysis of brewing attributes reveals that traditional methods do not fully capture the impact of the husk-separation in beer brewing. Therefore, conclusive empirical proof of the association between the established Kubessa technique and prolonged beer stability relies on innovative analytical approaches. Using ultrahigh resolution mass spectrometry (FT-ICR-MS) and machine learning algorithms, we demonstrated an aging-protective effect associated with over 500 aging-related compound signals. Additionally, Kubessa beer showed significant differences in its molecular profile, notably an increased presence of lipids containing sulfur, which are likely to include sulfonate groups. This aligns with the hypothesis that the Kubessa influences lipid metabolites in beer.^7^ Future studies employing specific brewing experiments and complementary methods will investigate potential molecular mechanisms and causal relationships beyond these correlations.

## Data Availability

The authors will make the FT-ICR-MS raw data available without undue reservation. The LC-ToF-MS data set was uploaded to the MassIVE (UC San Diego) environment (doi:10.25345/C5QB9VH2X).

## Supporting Information Available

Overview of the sample nomenclature; values for the established malt, wort and beer attributes; parameters for UPLC-separation and ToF-measurements; parameters of the UHPLC-ToF-MS data processing using the mzMine3 software; SIRIUS data processing and interpretation parameters; self-organizing map (SOM) clustering of FT-ICR-MS features based on intensity trends during beer aging; OPLS-DA statistics of the FT-ICR-MS data differentiating beers brewed with the Kubessa method (red) against whole-malt mashing (turquoise); statistical analysis of the UPLC-ToF-MS data differentiating beers brewed with the Kubessa method (red) against whole-malt mashing (turquoise); Tandem mass spectra of the compounds most specific for whole-grain mashing (VIP > 2.5) (PDF)
