# Supplementary material for: Husk Separation (Kubessa Method) Impacts the Aging Chemistry of Beer
Source: J Agric Food Chem. 2024 Sep 2;72(36):20048–55. doi: 10.1021/acs.jafc.4c05099 (PMC11403607; doi:10.1021/acs.jafc.4c05099)
Supplement: Supplementary file 1 — jf4c05099_si_001.pdf [file jf4c05099_si_001.pdf]

Supplementary information

Husk separation (Kubessa method) impacts the ageing chemistry of beer

Contents

Supplementary Table S1. Overview of the sample nomenclature. Every sample was created in triplicates (_R1 to _R3). 2

Supplementary Table S2. Values for the established malt, wort and beer attributes. 3

Supplementary Table S3. Parameters for UPLC-separation and ToF-measurements. 10

Supplementary Table S4. Parameters of the UHPLC-ToF-MS data processing using the mzMine3 software. 10

Supplementary Table S5. SIRIUS data processing and interpretation parameters. 11

Supplementary Figure S1. Self-organizing map (SOM) clustering of FT-ICR-MS features based on intensity trends during beer aging at time points t0 (finished beer), t1 (2 months), t2 (4 months), and t3 (6 months). 12

Supplementary Figure S2. OPLS-DA statistics of the FT-ICR-MS data with averaged triplicates differentiating beers brewed with the Kubessa method (red) against whole-malt mashing (turquoise). 13

Supplementary Figure S3. Statistical analysis of the UPLC-ToF-MS data in triplicates differentiating beers brewed with the Kubessa method (red) against whole-malt mashing (turquoise). 14

Supplementary Figure S4. Tandem mass spectra of the compounds most specific for whole-grain mashing (VIP>2.5). 15

Supplementary Tables

Supplementary Table S1. Overview of the sample nomenclature. Every sample was created in triplicates (_R1 to _R3).

Brewing/Aging step	Brewed/bottled at Distelhäuser brewery		Botteled at BLQ*	
	Kubessa mehod (K)	Whole-husk mashing (T)	K	T
Barley grain	A			
Barley endosperm	A_endosperm			
Barley husk	A_husk			
Mash	K_B	T_B		
Sweet wort	K_C	T_C		
Boiled wort	K_D	T_D		
Young beer	K_E	T_E		
Mature beer	D_K_t0	D_T_t0	K_F | B_K_t0	T_F | B_T_t0
Aged 2 month	D_K_t1	D_T_t1	B_K_t1	B_T_t1
Aged 4 month	D_K_t2	D_T_t2	B_K_t2	B_T_t2
Aged 7 month	D_K_t3	D_T_t3	B_K_t3	B_T_t3
Forced aging			B_K_fo	B_T_fo

* Research Center Weihenstephan for Brewing and Food Quality

Supplementary Table S2. Values for the established malt, wort and beer attributes.

Parameter	Unit	Values		
		**Malt** (average of three replicates)		
		whole malt	endosperm	husk
Aspartic acid	[mg/100 ml]	6.92	6.19	2.38
Glutamic acid	[mg/100 ml]	10.81	9.35	15.93
Asparagine	[mg/100 ml]	2.4	2.74	<0,50
Serine	[mg/100 ml]	4.2	4.09	3.67
Histidine	[mg/100 ml]	2.94	2.87	1.88
Glutamine	[mg/100 ml]	<0,50	32.2	10.56
Glycine	[mg/100 ml]	2.85	2.78	3.83
Threonine	[mg/100 ml]	5.65	5.35	5.65
Arginine	[mg/100 ml]	6.13	4.71	<0,50
Alanine	[mg/100 ml]	8.78	8.36	11.99
γ-Aminobutyric acid	[mg/100 ml]	5.84	6.05	2.11
Tyrosine	[mg/100 ml]	6.79	6.39	<0,50
Valine	[mg/100 ml]	9.18	8.84	6.08
Methionine	[mg/100 ml]	2.12	2	1.6
Tryptophan	[mg/100 ml]	2.67	2.37	1.09
Isoleucine	[mg/100 ml]	4.68	4.34	3.1
Phenylalanine	[mg/100 ml]	8.68	8.12	5.03
Leucine	[mg/100 ml]	9.73	9.32	7.44
Lysine	[mg/100 ml]	5.56	5.4	2.62
Amino acid content	[mg/100 ml]	105.94	131.36	85.33
FAN	[mg/100 ml]	11.9	14.7	9.5
Fructose	[g/l]	1.7	2.2	0.9
Glucose	[g/l]	4	3	0.9
Saccharose	[g/l]	2.4	1.2	0.2
Maltose	[g/l]	1.9	0.9	0.3
Maltotriose	[g/l]	<0,50	<0,50	<0,50
Water	[%]	4.8	4.9	4.9
Extract	[%]	18.1	19.6	11.4
Extract (dry matter)	[%]	19	20.6	12
Color	[EBC-Units]	2.7	2.6	2.6
Solble nitrogen	[g/ 100 g dm]	0.506	0.553	0.387
Thiobarbituric acid number	-	10.6	11.1	6.7
Thiobarbituric acid number (10)	-	13.2	13.9	8.4
pH-value	-	6.02	6.02	6.22

Parameter	Unit	Mash											
		K_B_R1	K_B_R2	K_B_R3	T1_B_R1	T1_B_R2	T1_B_R3	T2_B_R1	T2_B_R2	T2_B_R3	T3_B_R1	T3_B_R2	T3_B_R3
Specific gravity	-	1.07162	1.07748	1.07611	1.06891	1.07249	1.07456	1.07631	1.07994	1.09271	1.09311	1.07391	1.07628
Extract	[mas%]	17.42	18.76	18.45	16.8	17.62	18.09	18.49	19.31	22.17	22.26	17.95	18.48
pH-value	-	18.64	20.17	19.81	17.92	18.86	19.41	19.86	20.82	24.18	24.28	19.23	19.86

Color	[EBC-units]	6.7	6.6	6.4	6	6.3	6.4	6.9	7.1	8.3	7.3	6.4	6.7
Soluble nitrogen	[mg/100 ml]	158.2	173.6	172.2	163.8	177.8	177.8	179.2	184.8	212.8	205.8	175	182
Thiobarbituric acid number	-	28.8	30.6	29.8	27.7	28.6	29.5	29.5	30.7	34.9	32.1	28.8	30.2
Aspartic acid	[mg/100 ml]	4.31	5.46	5.98	5.56	5.64	5.93	5.27	5.09	4.91	4.04	4.45	4.33
Glutamic acid	[mg/100 ml]	5.77	6.54	7.04	5.86	5.24	5.59	5.57	5.53	5.54	5.43	4.84	6.06
Asparagine	[mg/100 ml]	7.65	7.4	8.02	6.95	6.3	6.68	6.59	5.98	6.55	7.52	6.05	5.36
Serine	[mg/100 ml]	5	5.74	6.22	6.06	4.67	5.07	5.82	5.56	5.79	5.03	5.16	4.68
Histidine	[mg/100 ml]	3.85	4.34	4.65	4.09	3.56	3.98	4.07	3.89	4.13	3.86	3.44	3.78
Glutamine	[mg/100 ml]	31.04	39.87	43.21	38.05	20.83	22.29	35.13	32.47	34.1	37.09	32.44	30
Glycine	[mg/100 ml]	2.49	2.93	3.13	3.03	1.78	2	2.95	2.79	3.08	2.59	2.69	2.87
Threonine	[mg/100 ml]	5.16	5.64	6.13	6.1	5	5.4	5.8	5.34	5.66	5.19	5.09	6.83
Arginine	[mg/100 ml]	11.47	12.42	13.59	12.81	11.09	11.79	12.14	11.5	12.28	11.88	10.92	6.61
Alanine	[mg/100 ml]	8.18	9.06	9.89	9.92	8.38	9.12	9.53	8.85	9.48	8.4	8.46	9.13
γ-Aminobutyric acid	[mg/100 ml]	5.94	6.38	6.73	7.33	4.76	5.34	7.08	6.46	7.42	6.22	6.23	5.74
Tyrosine	[mg/100 ml]	7.67	8.29	9.12	8.6	8.84	9.36	8.1	7.51	8.05	7.87	7.22	8.03
Valine	[mg/100 ml]	9.24	9.89	10.91	10.53	11.28	11.9	10.06	9.15	9.72	9.29	8.84	9.61
Methionine	[mg/100 ml]	2.78	3.03	3.3	3.38	3.02	3.26	3.19	2.92	3.15	2.9	2.85	3.04
Tryptophan	[mg/100 ml]	2.48	3.19	2.78	2.5	6.01	5.1	2.51	2.36	2.69	2.51	2.59	2.46
Isoleucine	[mg/100 ml]	5.4	5.99	6.92	6.79	8.36	8.73	6.3	5.82	5.99	5.45	5.4	5.88
Phenylalanine	[mg/100 ml]	10.52	11.81	13.04	12.16	14.34	14.85	11.41	10.72	11.23	10.56	10.08	11.2
Leucine	[mg/100 ml]	13.22	14.33	15.82	15.49	15.81	16.25	14.61	13.66	14.73	13.45	13.1	14.26
Lysine	[mg/100 ml]	7.99	9.35	9.55	9.36	8.77	9.61	8.74	8.42	9.13	7.66	7.96	8.63
Amino acid content	[mg/100 ml]	150.11	171.65	186.07	174.54	153.67	162.94	164.89	154	163.64	156.95	147.8	148.5
FAN	[mg/100 ml]	16.8	19.2	20.8	19.5	17.2	18.2	18.4	17.2	18.3	17.6	16.5	16.6
Fructose	[g/l]	2	2.1	2	2.4	2.4	2.4	2.8	2.4	1.8	2	2.5	2.2
Glucose	[g/l]	13.2	11.8	13.2	14	13.6	13.9	15.4	14.4	9.9	11.1	12.9	11.9
Saccharose	[g/l]	5	5.1	5	3.6	3.5	3.5	3.8	3.9	2.5	5	3.8	3.1
Maltose	[g/l]	81.8	86.3	82.3	77	72.2	75.8	86	88.9	64.4	64.9	77.2	65.5
Maltotriose	[g/l]	15.6	14.2	15.1	14.1	13.6	14.2	14.7	14.5	10.5	14.3	14.7	14.1
							Sweet wort						
		K_C_R1	K_C_R2	K_C_R3	T1_C_R1	T1_C_R2	T1_C_R3	T2_C_R1	T2_C_R2	T2_C_R3	T3_C_R1	T3_C_R2	T3_C_R3
Specific gravity	-	1.03797	1.04036	1.03502	1.04715	1.04836	1.04364	1.04185	1.03187	1.03938	1.03775	1.04211	1.04924
Extract	[mas%]	9.5	10.08	8.78	11.71	11.99	10.87	10.44	8.02	9.84	9.45	10.5	12.2
pH-value	-	5.34	5.32	4.73	5.44	5.37	5.57	5.51	5.23	5.45	5.52	5.41	5.44
Color	[EBC-units]	4.8	4.8	3.8	5.9	6	6.2	5.3	3.8	6.6	5.3	5.3	6.6
Soluble nitrogen	[mg/100 ml]	88.2	92.4	79.8	113.4	119	107.8	102.2	77	96.6	88.2	98	119
Thiobarbituric acid number	-	16.9	18.4	15.9	20.6	21.5	19.3	18	14	17.1	17.4	22.5	21.9
Aspartic acid	[mg/100 ml]	<0,50	1.41	4.59	5.11	3.95	<0,50	<0,50	<0,50	<0,50	<0,50	2.35	7.1
Glutamic acid	[mg/100 ml]	9.91	10.66	5.38	5.66	4.84	15.74	14.83	7.12	19.73	16.71	5.62	5.18
Asparagine	[mg/100 ml]	6.33	7.37	7.08	7.63	6.43	<0,50	<0,50	<0,50	0.67	<0,50	7.45	<0,50
Serine	[mg/100 ml]	2.9	1.02	5.11	5.68	4.94	<0,50	<0,50	<0,50	<0,50	<0,50	6.12	4.41
Histidine	[mg/100 ml]	4.31	n.d.	4.18	3.91	3.33	5.31	3.12	4.15	n.d.	n.d.	5.58	4.92
Glutamine	[mg/100 ml]	25	28.06	27.13	27.7	23.41	4.34	8.81	<0,50	10.05	<0,50	22.63	<0,50
Glycine	[mg/100 ml]	3.21	3.22	2.61	3.05	2.66	3.85	3.5	3.56	4.57	3.68	3.41	3.49
Threonine	[mg/100 ml]	6.61	6.34	5.12	6.08	5	7.24	7.73	6.44	8.87	6.02	7.76	6.88
Arginine	[mg/100 ml]	<0,50	11.57	12	12.04	11.08	12.35	<0,50	<0,50	1.01	<0,50	<0,50	<0,50
Alanine	[mg/100 ml]	10.17	10.14	8.33	9.56	8.35	13.85	12.87	10	15.06	11.24	12.69	11.49

γ-Aminobutyric acid	[mg/100 ml]	5.9	5.9	5.66	7.12	6.23	8.42	8.5	7.25	10.7	6.47	7.1	8.14
Tyrosine	[mg/100 ml]	8.91	8.76	8.12	8.55	7.2	10.6	10.62	9.02	11.75	8.76	11.39	9.98
Valine	[mg/100 ml]	12.15	12.49	10.42	10.63	8.82	14.07	14.26	11.49	16.41	11.4	13.84	12.75
Methionine	[mg/100 ml]	2.93	2.92	2.8	3.28	2.88	3.26	3.85	3.01	3.72	2.72	3.9	3.73
Tryptophan	[mg/100 ml]	2.6	2.56	2.5	2.36	2.38	3.5	3.88	2.88	5.72	3.89	4.32	3.37
Isoleucine	[mg/100 ml]	6.2	6.09	5.24	6.48	5.17	7.01	7.29	5.32	7.54	5.7	7.4	6.97
Phenylalanine	[mg/100 ml]	11.74	11.48	10.57	11.35	9.57	13.82	13.87	11.33	15.61	11.11	15.24	13.41
Leucine	[mg/100 ml]	14.31	14.23	13.51	14.68	13.19	17.12	17.8	14.13	19.31	13.97	19.24	17.07
Lysine	[mg/100 ml]	9.85	9.67	8.55	8.26	7.71	<0,50	10.6	11.49	14.08	<0,50	12.23	10.83
Amino acid content	[mg/100 ml]	143.46	154.16	148.9	159.11	137.15	141.02	142.38	107.48	165.08	103.24	168.42	130.61
FAN	[mg/100 ml]	16	17.2	16.7	17.8	15.3	15.8	15.9	12	18.5	11.5	18.8	14.6
Fructose	[g/l]	1	0.8	0.8	1.8	2.2	1.8	1.8	0.9	1.3	1.4	1.5	2
Glucose	[g/l]	6.4	6	6.5	8.8	9.9	9.5	8.9	6.2	6.5	7.1	8.4	9
Saccharose	[g/l]	2.8	2.6	2.9	2.1	2.6	2.2	2.2	1.7	2.4	2.3	2.1	2.5
Maltose	[g/l]	38.8	38.6	36.6	50	50.3	48	50.5	38.5	48.2	40.6	42.1	46.7
Maltotriose	[g/l]	8.3	8.5	8.4	15	14.7	15.3	12	8.4	11.8	9	9.55	11.8
Boiled wort													
		K_D_R1	K_D_R2	K_D_R3	T1_D_R1	T1_D_R2	T1_D_R3	T2_D_R1	T2_D_R2	T2_D_R3	T3_D_R1	T3_D_R2	T3_D_R3
Specific gravity	-	1.04532	1.03235	1.04304	1.04546	1.04523	1.0541	1.03988	1.04347	1.03884	1.06014	1.05428	1.04478
Extract	[mas%]	11.27	8.13	10.72	11.36	11.25	13.35	9.96	10.83	9.71	14.77	13.4	11.14
pH-value	-	5.28	5.33	5.26	5.38	5.34	5.31	5.38	5.23	5.37	5.31	5.31	5.4
Color	[EBC-units]	6	4.6	6.1	6.3	6.3	7.5	6.3	6.7	5.5	8.2	7.9	6.3
Soluble nitrogen	[mg/100 ml]	95.2	70	93.8	102.2	103.6	124.6	91	96.6	86.8	131.6	123.2	102.2
Thiobarbituric acid number	-	31.8	25	28.8	33.6	32.3	40.1	24.9	30	25.4	40	37.8	32.7
Aspartic acid	[mg/100 ml]	3.72	<0,50	5.03	6.75	4.06	<0,50	<0,50	2.74	6.23	<0,50	1.79	5
Glutamic acid	[mg/100 ml]	4.58	11.93	5.06	6.5	3.74	0.8	8.73	6.87	6.06	3.5	5.08	5.32
Asparagine	[mg/100 ml]	9.61	7.5	9.29	5.15	6.93	<0,50	5.49	8.13	10.87	<0,50	8.3	9.33
Serine	[mg/100 ml]	5.92	<0,50	6.03	5.89	4.87	<0,50	<0,50	3.71	7.7	<0,50	3.92	6.38
Histidine	[mg/100 ml]	4.33	6	4.24	4.58	3.54	n.d.	5.53	4.32	5.78	n.d.	3.28	4.7
Glutamine	[mg/100 ml]	10.63	10.5	9.35	10.73	8.76	3.39	7.44	5.72	12.21	<0,50	7.13	11.61
Glycine	[mg/100 ml]	3.23	4.61	3.02	3.52	2.41	6.2	4.26	3.91	3.92	2.25	2.75	3.43
Threonine	[mg/100 ml]	5.87	9.27	5.69	9.4	4.89	<0,50	8.49	6.75	7.93	5.12	5.26	7.97
Arginine	[mg/100 ml]	10.48	2.44	11.64	<0,50	10.02	<0,50	<0,50	10.36	14.93	<0,50	11.16	9.18
Alanine	[mg/100 ml]	10.38	14.64	9.98	11.22	8.24	10.89	13.63	11.69	13.41	7.91	8.9	11.66
γ-Aminobutyric acid	[mg/100 ml]	6.74	9.42	6.45	7.94	5.8	10.72	9.09	7.07	9.97	6.36	6.78	7.89
Tyrosine	[mg/100 ml]	7.85	12.39	7.91	10	7.08	8.93	10.84	8.94	11.67	7.13	7.5	9.32
Valine	[mg/100 ml]	11.41	16.65	11.32	12.25	9.52	12.18	14.72	12.86	15.24	9.45	9.5	12.9
Methionine	[mg/100 ml]	2.93	3.69	3	3.72	2.67	2.5	3.59	3.24	4.28	2.21	3.01	3.65
Tryptophan	[mg/100 ml]	3	5.16	3	3.47	2.64	4.62	4.31	3.17	4.67	2.86	1.93	3.44
Isoleucine	[mg/100 ml]	5.74	7.91	5.7	7.22	5.15	6.68	7.65	6.06	8.37	4.29	4.94	6.74
Phenylalanine	[mg/100 ml]	11.12	17.01	11.02	13.13	9.74	13.15	15.64	11.62	16.01	9.29	9.14	12.7
Leucine	[mg/100 ml]	13.94	19.81	14.15	16.84	12.36	15.9	20.09	15.12	19.99	13.08	13.63	16.62
Lysine	[mg/100 ml]	8.29	7.69	8.44	9.71	7.51	<0,50	14.79	1.51	7.07	8.42	6.88	2.67
Amino acid content	[mg/100 ml]	139.75	166.92	140.32	148.34	119.96	97.04	154.92	133.8	186.3	82.84	121	150.51
FAN	[mg/100 ml]	15.6	18.7	15.7	16.6	13.4	10.9	17.3	15	20.8	9.3	13.5	16.8
Fructose	[g/l]	1.2	2.5	2.6	2.9	2.9	2.2	2.6	2.7	2.7	2.6	2.6	2.6
Glucose	[g/l]	8.8	8.4	8.3	8.6	9.1	7.1	8.9	8	8.7	7.1	6.9	8.4

Saccharose	[g/l]	3.5	2.9	3.2	2.9	3	2.3	2.8	2.9	2.7	2.3	2.4	2.7
Maltose	[g/l]	51	50.2	53.7	60.4	51.4	34.6	53.1	54.3	52.8	34.8	37.4	57.5
Maltotriose	[g/l]	11	11.8	15	15.2	15.4	10.7	14.6	15	14.7	10.8	10.3	15.3
Cooled wort													
		K_E_R1	K_E_R2	K_E_R3	T1_E_R1	T1_E_R2	T1_E_R3	T2_E_R1	T2_E_R2	T2_E_R3	T3_E_R1	T3_E_R2	T3_E_R3
Specific gravity	-	1.04289	1.04051	1.04309	1.04256	1.04332	1.04766	1.04396	1.04494	1.0442	1.04747	1.04383	1.04296
Extract	[mas%]	10.69	10.12	10.73	10.61	10.79	11.83	10.94	11.18	11	11.78	10.91	10.7
pH-value	-	5.21	5.21	5.2	5.29	5.27	5.26	5.27	5.25	5.26	5.28	5.29	5.29
Color	[EBC-units]	6.9	6.9	6.8	8	7.8	8.2	7.9	8	8.3	7.9	7.4	7.9
Soluble nitrogen	[mg/100 ml]	91	86.8	92.4	93.8	98	106.4	98	98	98	102.2	98	96.6
Thiobarbituric acid number	-	35.7	37.2	35.9	39.8	41	43.5	38.7	43.9	42.9	41.2	37.7	37.1
Aspartic acid	[mg/100 ml]	4.98	1.27	1.28	4.19	3.26	1.32	6.48	4.27	4.58	2.29	0.6	3.95
Glutamic acid	[mg/100 ml]	5.71	8.23	3.15	4.43	4.3	2.01	4.89	3.61	4.75	6.29	5.35	4.72
Asparagine	[mg/100 ml]	9.66	8.42	8.93	9.06	9.67	6.09	9.08	8.38	10.39	7.71	8.43	8.19
Serine	[mg/100 ml]	5.55	1.76	5.09	6.88	5.32	3.81	6.72	6.1	7.73	4.48	6.91	5.86
Histidine	[mg/100 ml]	4.87	n.d.	4.95	4.81	3.62	3.18	5.01	3.85	5.14	4.59	4.93	5.11
Glutamine	[mg/100 ml]	7.56	2.09	7.57	2.8	3.64	2.83	3.89	2.17	3.18	3.6	2.02	4.15
Glycine	[mg/100 ml]	2.72	3.02	2.69	3.56	2.93	2.03	3.39	3.24	4.65	3.16	4.01	3.31
Threonine	[mg/100 ml]	6.11	7.22	7.78	6.05	5.37	4.15	6.79	5.19	8.78	7.04	7.94	8.01
Arginine	[mg/100 ml]	12.26	3.77	2.25	13.03	11.67	6.15	12.83	10.45	6.25	7.77	1.15	3.4
Alanine	[mg/100 ml]	9	11.45	9.41	11.36	9.11	6.83	11.56	10.24	14.17	9.89	13.35	10.87
γ-Aminobutyric acid	[mg/100 ml]	6.35	7.42	5.07	8.41	7.08	4.2	8.11	7.42	9.48	6.9	8.48	7.8
Tyrosine	[mg/100 ml]	9.37	9.74	9.7	8.57	7.67	6.28	9.73	7	8.95	8.36	8.97	10.1
Valine	[mg/100 ml]	11.36	12.47	11.47	10.5	9.67	7.83	13.01	10.07	13.59	9.81	11.66	12.51
Methionine	[mg/100 ml]	2.85	2.86	3.05	3.17	3	2.11	3.53	2.81	3.72	2.9	3.33	3.53
Tryptophan	[mg/100 ml]	3.74	5.15	3.74	3	2.38	2	3.57	2.49	3.43	3.08	2.62	3.73
Isoleucine	[mg/100 ml]	6.14	7.95	5.91	5.85	5.01	3.91	6.95	5.09	7.01	5.24	5.42	6.81
Phenylalanine	[mg/100 ml]	12.53	16.24	12.67	11.18	9.46	7.73	13.16	9.47	13.19	10.98	10.84	13.29
Leucine	[mg/100 ml]	14.62	18.38	15.01	16.24	14.29	10.44	16.81	13.55	17.82	14.77	17.39	16.83
Lysine	[mg/100 ml]	9.58	12.77	9.98	9.79	7.7	6.52	10	8.28	10.63	8.78	8.82	10.45
Amino acid content	[mg/100 ml]	144.93	140.56	129.68	142.83	125.14	89.43	155.51	123.69	157.44	127.62	132.21	142.61
FAN	[mg/100 ml]	16.2	15.7	14.5	16	14	10	17.4	13.8	17.6	14.3	14.8	16
Fructose	[g/l]	1.5	1.2	1.4	1.2	0.6	1.2	2.8	1.4	1.1	1.8	1.2	1.7
Glucose	[g/l]	8.8	7.5	8.3	6.5	4	5.6	9.2	7.6	6.2	9.1	5.7	8.3
Saccharose	[g/l]	3.2	3	3.2	1.8	1	1.4	2.9	2.2	1.7	2.5	1.7	2.3
Maltose	[g/l]	54.5	54.4	53.4	41.3	20.9	27	55.7	49.6	44.6	55.5	29.7	51
Maltotriose	[g/l]	11.7	10.6	11.4	8.3	5	6.6	14.9	10.3	10	12.7	6.5	13.1

Mature beer and forced aged beer									
		B_K_t0	B_T_t0_R1	B_T_t0_R2	B_T_t0_R3	B_K_fo	B_T_fo_R1	B_T_fo_R2	B_T_fo_R3
Original gravity	[mas%]	11.86	11.81	11.88	11.97				
Original gravity	[mas/vol%]	12.41	12.35	12.42	12.52				
Alcohol content	[mas%]	3.93	3.9	3.91	3.93				
Alcohol content	[vol%]	5.01	4.98	5	5.02				
Apparent extract	[mas%]	2.43	2.44	2.48	2.53				
Apparent extract	[mas/vol%]	2.45	2.46	2.5	2.55				
Specific gravity	-	1.00949	1.00952	1.00966	1.00989				
Real extract	[mas%]	4.25	4.24	4.29	4.35				

Real extract	[mas/vol%]	4.31	4.31	4.35	4.42				
Apparent attenuation	[%]	80.3	80.1	79.9	79.6				
Real attenuation	[%]	64.2	64.1	63.9	63.6				
pH-value	-	4.55	4.53	4.53	4.55				
DLG score (odor)	-	4.4	4.3	4.4	4.3	3.4	3.8	3.2	3.4
DLG taste/aroma		-	-	-	-	aged, sherrylike	aged	aged, sherrylike	aged, sherrylike
DLG score (taste)	-	4.4	4.3	4.4	4.3	3.4	3.8	3.2	3.4
DLG off taste/aroma		-	-	-	-	aged, sherrylike	aged	aged, sherrylike	aged, sherrylike
DLG score (palate fullness)	-	4.6	4.6	4.6	4.6	4.6	4.6	4.6	4.4
DLG score (effervescence)	-	4.6	4.6	4.6	4.6	4.6	4.6	4.6	4.6
DLG score (quality of bitterness)	-	4.2	4.0	4.3	4.2	3.2	3.6	3.6	3.5
DLG (flavor stability)	-					0.75	0.35	0.78	0.61
DLG score (overall)	-	4.40	4.30	4.43	4.30	3.65	3.95	3.65	3.70
2-Methylbutanal (O/A)	[µg/l]	<5	<5	<5	<5	8	7	<5	<5
3-Methylbutanal (O/A)	[µg/l]	14	18	15	10	26	23	14	13
2-Furfural (H/A)	[µg/l]	<5	<5	<5	<5	95	79	70	67
5-Methylfurfural (A)	[µg/l]	<5	<5	<5	<5	<5	<5	<5	<5
Benzaldehyde (O,A)	[µg/l]	<5	<5	<5	<5	<5	<5	<5	<5
2-Phenylethanal (O,A)	[µg/l]	11	16	11	<5	25	23	11	6
Diethylsuccinate (A)	[µg/l]	<5	<5	<5	<5	<5	<5	<5	<5
Ethyl nicotinate (H/A)	[µg/l]	<5	<5	<5	<5	9	8	8	8
Ethyl phenylacetat (A)	[µg/l]	<5	<5	<5	<5	<5	<5	<5	<5
2-Acetylfuran (A)	[µg/l]	11	11	10	11	11	11	12	11
2-Propionyl-furan (A)	[µg/l]	7	7	6	7	7	7	7	7
γ-Nonalacton (H/A)	[µg/l]	22	21	19	18	29	26	24	26
Sum of heat indicators (H)	[µg/l]	22	21	19	18	133	112	102	100
Sum of oxygen indicators (O)	[µg/l]	25	34	26	10	59	52	24	19
Sum of aging indicators (A)	[µg/l]	64	71	60	46	210	181	145	135
Ethyl butyrate	[mg/l]	0.07	0.05	0.06	0.06				
Isobutyl acetate	[mg/l]	0.04	0.03	0.04	0.04				
Phenylethyl acetate	[mg/l]	0.37	0.31	0.34	0.29				
Ethyl hexanoate	[mg/l]	0.12	0.1	0.1	0.1				
Ethyl octanoate	[mg/l]	0.22	0.19	0.19	0.19				
Ethyl caprate	[mg/l]	0.08	0.06	0.07	0.07				
Isovaleric acid	[mg/l]	1.53	1.55	1.67	1.61				
Caproic acid	[mg/l]	1.92	1.51	1.58	1.67				
Caprylic acid	[mg/l]	2.66	2.11	1.99	2.41				
Capric acid	[mg/l]	0.44	0.34	0.32	0.4				
Acetaldehyde	[mg/l]	10	9.8	10.7	9.7				
Ethylacetate	[mg/l]	11.4	12	12	10.7				
Propyl alcohol	[mg/l]	9.6	10.7	10	9.9				
2-Methylpropanol	[mg/l]	10.3	11.4	10.7	10.7				
Isoamyl acetate	[mg/l]	1	1	1.1	0.9				
Amyl alcohols	[mg/l]	66.9	68.2	67.5	65.3				
Butan-2,3-dione (Diacetyl)	[mg/l]	0.02	0.02	0.02	0.02				
Pentan-2,3-dione	[mg/l]	<0,01	<0,01	<0,01	<0,01				
Acetoin	[mg/l]	4	4.9	4.2	4.4				
Dimethylsulfide	[µg/l]	66	75	70	76				

2-Phenylethanol	[mg/l]	21.9	18	18.1	15.4
SO_2	[mg/l]	3	3	3	3
Color	[EBC-units]	5.2	5.2	5.2	5.2
Fructose	[g/l]	0.1	0.3	0.1	0.3
Glucose	[g/l]	0.3	0.4	0.2	0.4
Saccharose	[g/l]	<0,10	<0,10	<0,10	<0,10
Maltose	[g/l]	1	1.2	1	0.9
Maltotriose	[g/l]	2.4	1.6	2.6	2
Aspartic acid	[mg/100 ml]	5.21	5.46	5.32	5.93
Glutamic acid	[mg/100 ml]	5.67	5.26	5.07	5.81
Asparagine	[mg/100 ml]	3.65	3.83	3.57	4.39
Serine	[mg/100 ml]	1.34	1.6	1.45	1.67
Histidine	[mg/100 ml]	4.71	4.75	4.72	4.82
Glutamine	[mg/100 ml]	4.62	4.55	4.48	3.76
Glycine	[mg/100 ml]	4.84	4.65	5.17	5.02
Threonine	[mg/100 ml]	1.38	1.53	1.47	1.69
Arginine	[mg/100 ml]	11.78	12.43	12.39	11.8
Alanine	[mg/100 ml]	15.25	14.5	14.78	14.92
γ-Aminobutyric acid	[mg/100 ml]	11.3	12.31	12.19	11.41
Tyrosine	[mg/100 ml]	9.59	11.14	10.05	10.04
Valine	[mg/100 ml]	10.28	11.63	11.23	11.39
Methionine	[mg/100 ml]	1.29	1.6	1.49	1.61
Tryptophan	[mg/100 ml]	3.37	3.22	3.37	3.24
Isoleucine	[mg/100 ml]	3.88	4.78	4.56	4.72
Phenylalanine	[mg/100 ml]	10.47	12.13	11.33	11.76
Leucine	[mg/100 ml]	6.28	7.69	7.39	7.94
Lysine	[mg/100 ml]	4.09	4.78	4.74	5
Amino acid content	[mg/100 ml]	119	127.83	124.77	126.9
FAN	[mg/100 ml]	146.4	157.2	153.5	156.1
Soluble nitrogen	[mg/100 ml]	83	86.1	85.4	85.8
Thiobarbituric acid number	-	34.1	34.3	33.8	35.2

Aging experiment													
		B_K_t1	B_T_t1_R1	B_T_t1_R2	B_T_t1_R3	B_K_t2	B_T_t2_R1	B_T_t2_R2	B_T_t2_R3	B_K_t3	B_T_t3_R1	B_T_t3_R2	B_T_t3_R3
DLG score (odor)	-	4.2	4.2	4.3	4.3	4.1	4.0	3.5	3.8	3.2	3.8	3.6	4.0
DLG taste/aroma		-	-	-	-	-	slightly aged, traces of spent grain aroma	aged, carbord	aged	aged, breadlike	aged	aged, breadlike	-
DLG score (taste)	-	4.2	4.2	4.2	4.3	4.1	4.0	3.5	3.8	3.4	3.9	3.6	4.0
DLG off taste/aroma		-	-	-	-	-	slightly aged, traces of spent grain aroma	aged, carbord	aged	aged, breadlike	aged	aged, breadlike	-
DLG score (palate fullness)	-	4.6	4.6	4.6	4.6	4.6	4.6	4.6	4.6	4.5	4.5	4.5	4.5
DLG score (effervescence)	-	4.6	4.6	4.6	4.6	4.6	4.6	4.6	4.6	4.5	4.5	4.5	4.5
DLG score (quality of bitterness)	-	4.3	4.2	4.3	4.3	3.9	3.7	3.5	3.7	3.8	3.9	3.8	3.8
DLG score (overall)	-	4.33	4.30	4.35	4.38	4.18	4.08	3.78	3.98	3.73	4.03	3.88	4.08
2-Methylbutanal (O/A)	[µg/l]	<5	<5	<5	<5	13	16	13	14	<5	6	<5	<5
3-Methylbutanal (O/A)	[µg/l]	13	11	9	6	13	17	15	14	17	19	18	11
2-Furfural (H/A)	[µg/l]	<5	9	<5	<5	5	6	8	<5	237	236	234	237

5-Methylfurfural (A)	[µg/l]	<5	<5	<5	<5	<5	<5	<5	<5	102	112	100	104
Benzaldehyde (O,A)	[µg/l]	<5	<5	<5	<5	<5	<5	<5	<5	87	81	84	83
2-Phenylethanal (O,A)	[µg/l]	10	5	<5	<5	6	5	12	<5	<5	<5	<5	<5
Diethylsuccinate (A)	[µg/l]	<5	<5	<5	<5	<5	<5	<5	<5	<5	<5	<5	<5
Ethyl nicotinate (H/A)	[µg/l]	<5	<5	<5	<5	<5	<5	<5	<5	<5	<5	<5	<5
Ethyl phenylacetat (A)	[µg/l]	<5	<5	<5	<5	<5	<5	<5	<5	<5	<5	<5	<5
2-Acetylfuran (A)	[µg/l]	9	9	9	10	9	9	8	9	9	8	9	9
2-Propionyl-furan (A)	[µg/l]	6	5	6	6	5	6	5	6	5	5	5	5
γ-Nonalacton (H/A)	[µg/l]	16	17	17	17	24	23	26	34	26	28	27	25
Sum of heat indicators (H)	[µg/l]	16	26	17	17	29	29	33	34	263	264	261	262
Sum of oxygen indicators (O)	[µg/l]	23	16	9	6	31	38	40	28	104	105	103	94
Sum of aging indicators (A)	[µg/l]	54	55	41	39	74	81	87	77	483	494	477	475
Ethyl butyrate	[mg/l]	0.06	0.06	0.06	0.07	0.06	0.05	0.06	0.06	0.05	0.06	0.06	0.06
Isobutyl acetate	[mg/l]	n.d.	n.d.	n.d.	n.d.	0.03	0.03	0.03	0.03	0.03	0.03	0.03	0.02
Phenylethyl acetate	[mg/l]	0.32	0.26	0.29	0.25	0.37	0.28	0.32	0.28	0.06	0.27	0.31	0.26
Ethyl hexanoate	[mg/l]	0.1	0.08	0.08	0.09	0.11	0.09	0.09	0.09	0.1	0.09	0.09	0.09
Ethyl octanoate	[mg/l]	0.21	0.18	0.18	0.2	0.23	0.2	0.18	0.18	0.21	0.11	0.19	0.18
Ethyl caprate	[mg/l]	0.06	0.05	0.06	0.05	0.01	0.01	0.03	0.03	0.04	0.05	0.07	0.03
Isovaleric acid	[mg/l]	1.12	1.16	1.1	1.25	1.07	1.03	1.12	1.02	1.07	0.97	0.91	1.01
Caproic acid	[mg/l]	1.94	1.37	1.49	1.61	2.41	1.81	1.85	1.88	2.37	2.21	1.92	1.99
Caprylic acid	[mg/l]	3.21	2.48	2.67	2.69	3.41	3.41	2.56	2.74	2.75	2.07	2.77	2.77
Capric acid	[mg/l]	0.27	0.17	0.22	0.2	0.43	0.39	0.32	0.3	0.39	0.44	0.41	0.41
Acetaldehyde	[mg/l]	10.2	8.9	9.7	9.1	8.9	8.3	9.3	9.1	9.4	9.3	10.3	9.9
Ethylacetate	[mg/l]	12.2	12.1	12.4	11.1	11.2	10.6	11.4	10.6	11.6	11.7	12.3	11.7
Propyl alcohol	[mg/l]	9.5	10.6	10	10.1	9	10	9.5	9.6	9.5	10.6	10.2	10.4
2-Methylpropanol	[mg/l]	10.7	11.4	10.8	10.9	9.8	10.3	10.3	10.2	10.3	11	11	11.2
Isoamyl acetate	[mg/l]	1.1	1	1.1	0.9	1	0.9	1	0.9	1	0.9	1.1	1
Amyl alcohols	[mg/l]	69.3	70.4	68.8	66.6	64.3	63.5	64.8	62.4	66.8	68.2	68.9	68.3
Butan-2,3-dione (Diacetyl)	[mg/l]	0.05	0.05	0.04	0.05	0.04	0.06	0.05	0.07	0.06	0.1	0.07	0.08
Pentan-2,3-dione	[mg/l]	0.01	0.01	0.01	0.01	<0,01	0.02	0.01	0.02	0.02	0.03	0.02	0.02
Acetoin	[mg/l]	4.4	5.4	4.6	5	2.9	3.4	3	3.2	3.1	3.6	3.2	3.4
Dimethylsulfide	[µg/l]	40	43	40	47	68	72	71	73	58	66	63	65
2-Phenylethanol	[mg/l]	19.2	17.1	17	17	22.9	17.4	19.7	16.5	18.1	17.8	18.3	18.3

Supplementary Table S3. Parameters for UPLC-separation and ToF-measurements.

Parameter	Value
SPE sample preparation	doi.org/10.3389/fchem.2021.715372
column	RP (C18: 1.7 µm, 2.1 x 100 mm, Acquity^{TM} UPLC BEH^{TM})
flow rate	400 µL min^{-1}
column temperature	40 °C
injection volume	5 µL
gradient profile	95 % A (0.1 % formic acid in water) and 5 % B (0.1 % formic acid in acetonitrile) for 1.2 min; decreasing to 50 % A in 2.5 min; decreasing to 0.5 % A in 7.8 min; held for 3.5 min; equilibrated in starting conditions for 3.5 min.
measurement time	18.5 min.
external calibration	ESI negative calibration solution (SCIEX X500B System)
ESI ionization mode	negative
Ion source gas	45 psi
Curtain gas	45 psi
interface temperature	500°C
MS^1 parameters	0.496 sec^{-1} event cycle time
	50-1500 Da mass range
	Accumulation time 0.2 sec
MS^2 fragmentation parameters	DDA (8 dependent events)
	Accumulation time 0.03 sec
	Time bins to sum 4
	CE spread 30 eV ± 15 eV

Supplementary Table S4. Parameters of the UHPLC-ToF-MS data processing using the mzMine3 software.

Parameter	value
MS1 | MS2 noise level	800 | 50
Minimum peak height	1800
Minimum peak width	5 scans
m/z tolerance	0.005 Da or 10 ppm
Smoothing	Savitzky Golay (7)
Local minimum resolution	chrom. threshold 0.9
	Min ratio peak top / edge 2.0
13C isotope filter	applied
Peak alignment	0.005 Da or 10 ppm
	9 sec. RT tolerance
	m/z weight 3 | RT weight 2
MFG export (SIRIUS): MS/MS merge	0.01 Da or 20 ppm
	Cosine threshold 0.65 %
	Signal count threshold 33 %

Supplementary Table S5. SIRIUS data processing and interpretation parameters.

Parameter	value
SIRIUS Molecular formula identification	$C_{\infty}H_{\infty}$ N_{∞} O_{∞} S_3 P_3<br>Mass accuracy 5 ppm
CSI:FingerID Fingerprint Prediction	$[M-H]^-$ $[M+Cl]^-$ $[M+Br]^-$
CSI:FingerID Structure Database Search	Bio Database, Biocyc, CHEBI, COONUT, EcoCyc Mine, GNPS, HMDB, HSDB, KEGG, KEGG Mine, KNApSAcK, Maconda, MESH, NORMAN, Natural Products, Plantcyc, PubChem, PubMed, YMDB, YMDB Mine, ZINC bio
CANOPUS Compound Class Prediction	Subclass level

Supplementary Figures

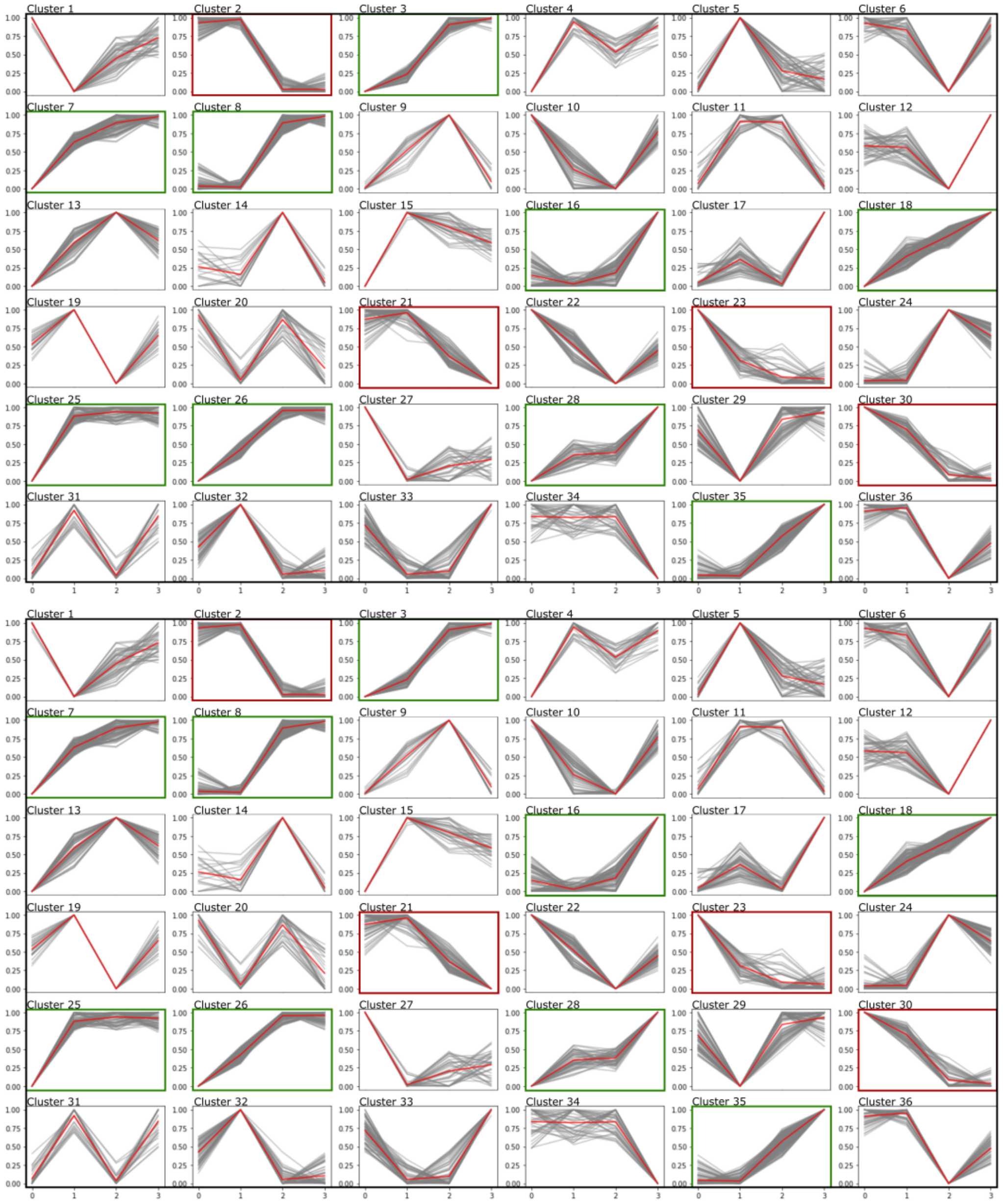

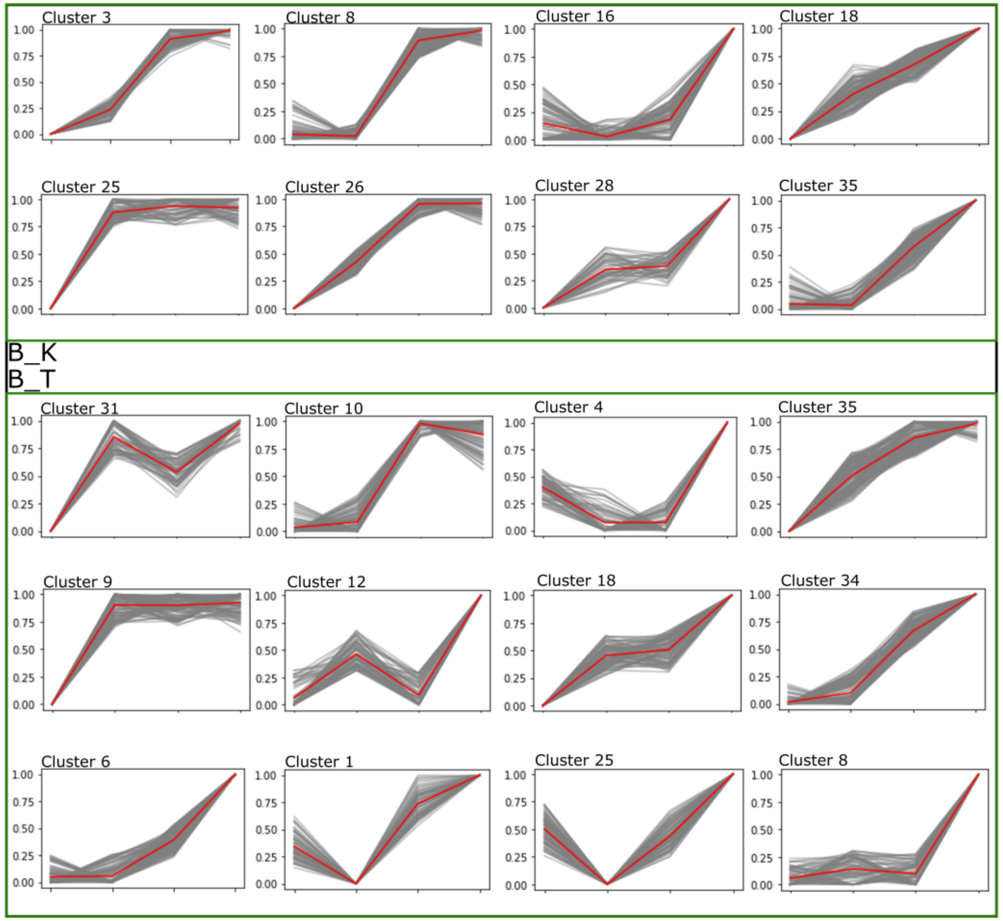

Supplementary Figure S1. Self-organizing map (SOM) clustering of FT-ICR-MS features based on intensity trends during beer aging at time points t0 (finished beer), t1 (2 months), t2 (4 months), and t3 (6 months).
Increasing trends are highlighted in green, decreasing trends in red. SOM analysis conducted separately for Kubessa method (top left) and whole-malt mashing (bottom left), with increasing trends isolated on the right. Features present in Clusters 3, 8, 16, 18, 25, 26, 28, or 35 for Kubessa (B_K), and at the same time in Clusters 31, 10, 4, 35, 9, 12, 18, 34, 6, 1, 25, 8 for whole-malt mashing (B_T), were identified as aging-related.

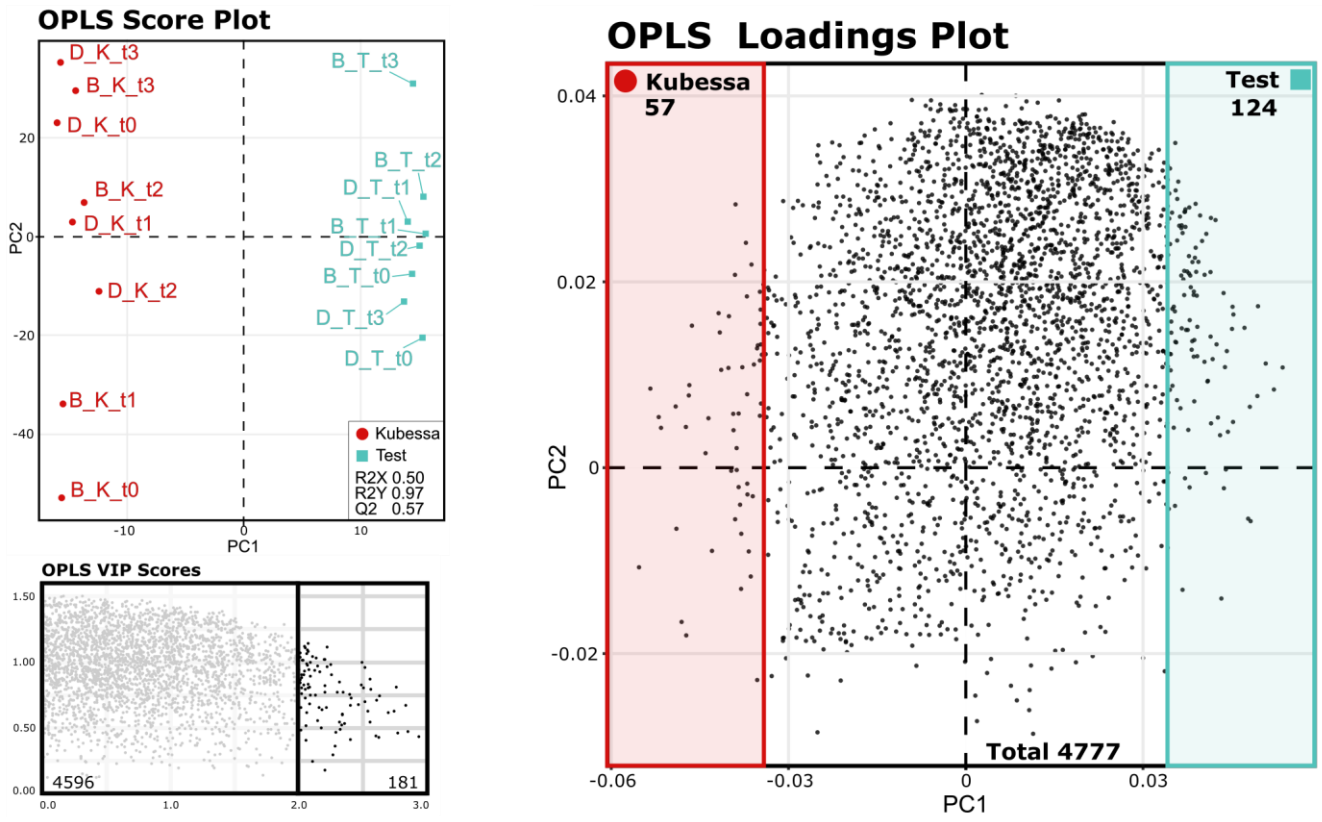

Supplementary Figure S2. OPLS-DA statistics of the FT-ICR-MS data with averaged triplicates differentiating beers brewed with the Kubessa method (red) against whole-malt mashing (turquoise).
In the score plot (top left), samples are distinctly differentiated. Features with VIP values greater than 2 were identified as particularly significant (bottom left) and are highlighted in the corresponding loadings plot (right).

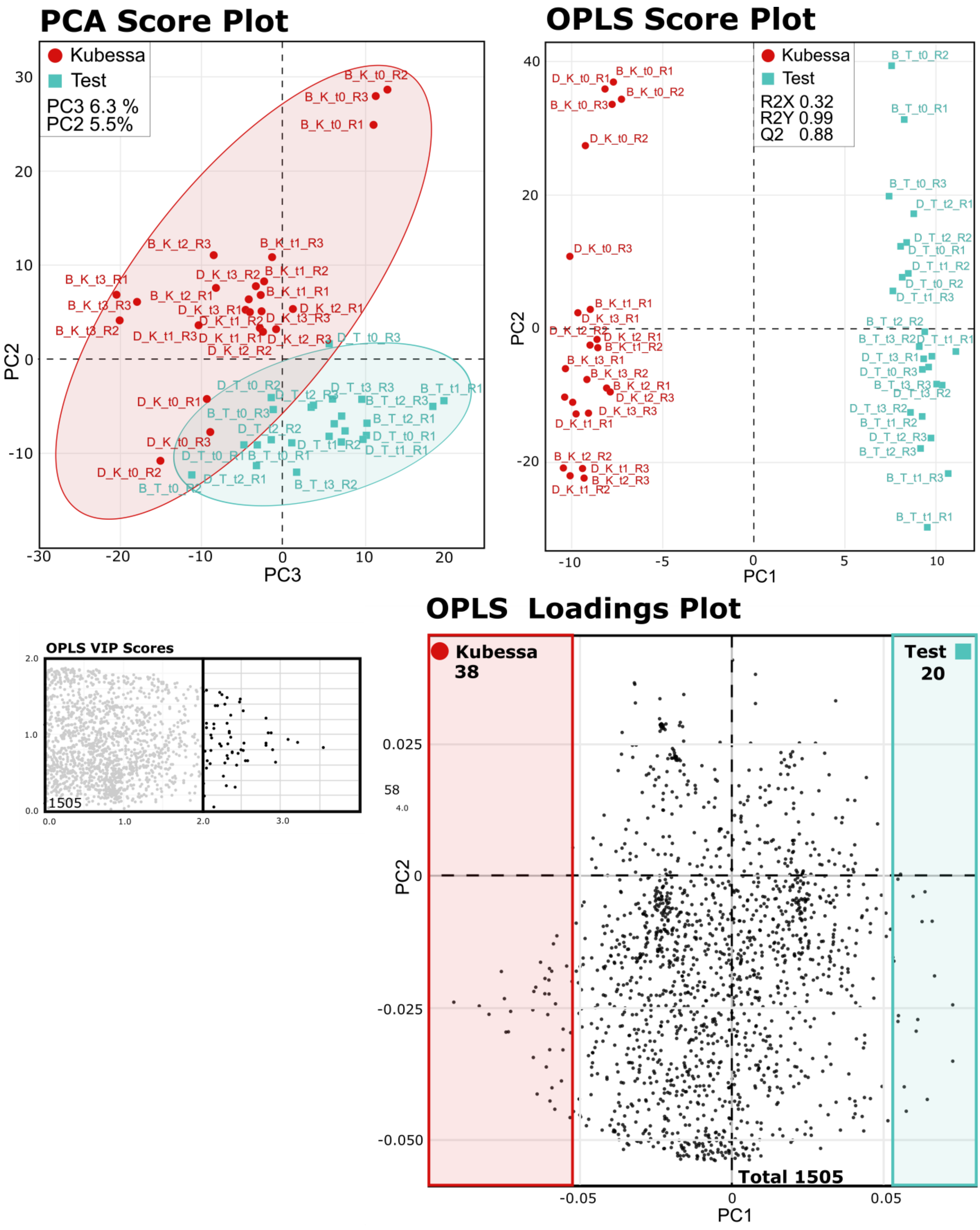

Supplementary Figure S3. Statistical analysis of the UPLC-ToF-MS data in triplicates differentiating beers brewed with the Kubessa method (red) against whole-malt mashing (turquoise).
In the score plot of the unsupervised PCA (top left), the samples are already diverging. The OPLS-DA clearly differentiates the brewing lines (top right). Features with VIP values greater than 2 were identified as particularly significant (bottom left) and are highlighted in the corresponding loadings plot (bottom right).

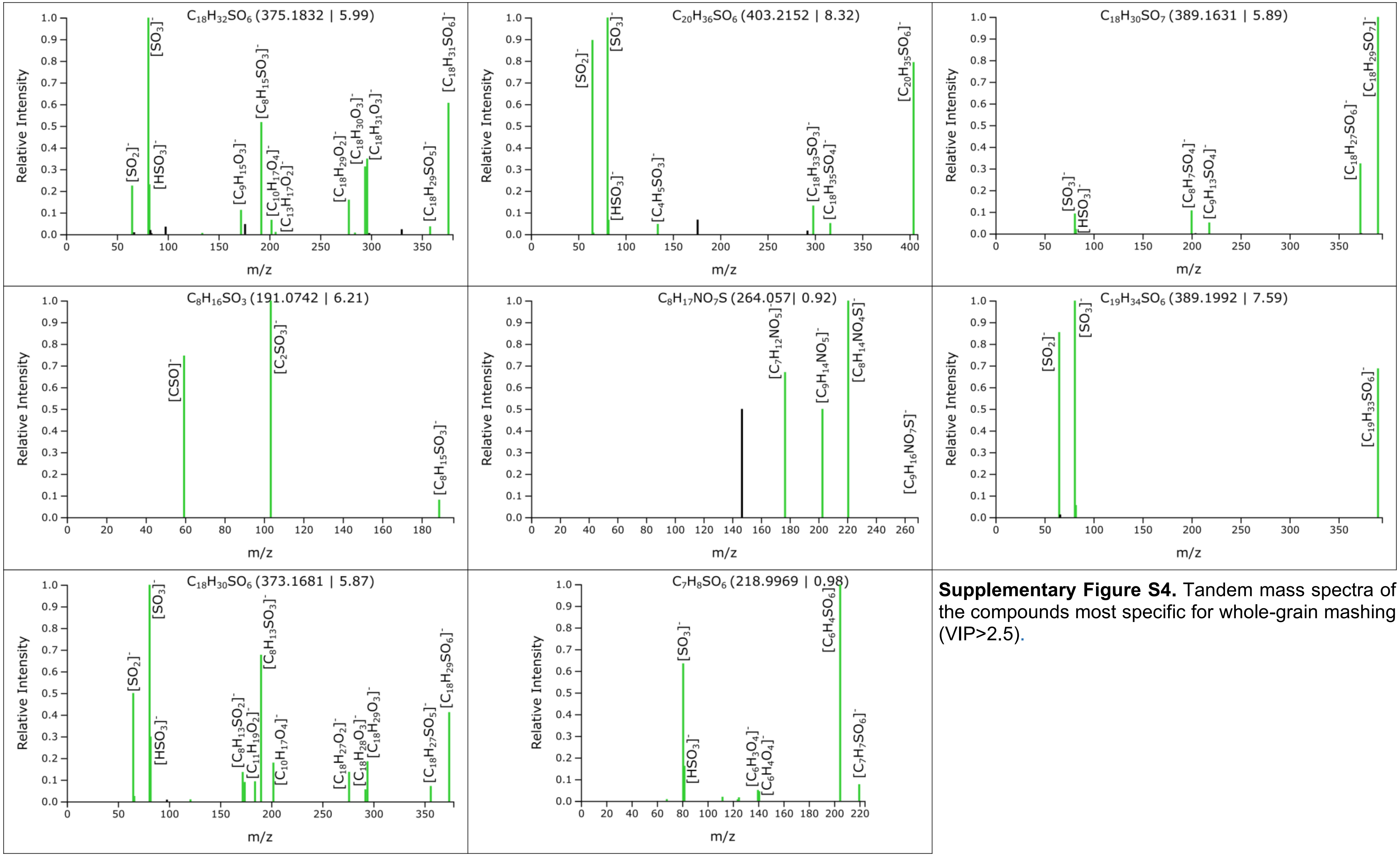

Supplementary Figure S4. Tandem mass spectra of the compounds most specific for whole-grain mashing (VIP>2.5).
